# MPA alters metabolic phenotype of endometrial cancer-associated fibroblasts from obese women via IRS2 signaling

**DOI:** 10.1371/journal.pone.0270830

**Published:** 2022-07-11

**Authors:** Intan Sofia Omar, Amira Hajirah Abd Jamil, Noor Azmi Mat Adenan, Ivy Chung

**Affiliations:** 1 Department of Pharmacology, Faculty of Medicine, Universiti Malaya, Kuala Lumpur, Malaysia; 2 Universiti Malaya Cancer Research Institute, Faculty of Medicine, Universiti Malaya, Kuala Lumpur, Malaysia; 3 Department of Pharmaceutical Life Sciences, Faculty of Pharmacy, Universiti Malaya, Kuala Lumpur, Malaysia; 4 Department of Obstetrics and Gynaecology, Faculty of Medicine, Universiti Malaya, Kuala Lumpur, Malaysia; 5 Department of Obstetrics and Gynaecology, Ara Damansara and Subang Jaya Medical Center, Ramsay Sime Darby Health Care, Subang Jaya, Selangor, Malaysia; Université de Bourgogne: Universite de Bourgogne, FRANCE

## Abstract

Obese women have a higher risk of developing endometrial cancer (EC) than lean women. Besides affecting EC progression, obesity also affects sensitivity of patients to treatment including medroxprogesterone acetate (MPA). Obese women have a lower response to MPA with an increased risk for tumor recurrence. While MPA inhibits the growth of normal fibroblasts, human endometrial cancer-associated fibroblasts (CAFs) were reported to be less responsive to MPA. However, it is still unknown how CAFs from obese women respond to progesterone. CAFs from the EC tissues of obese (CO) and non-obese (CN) women were established as primary cell models. MPA increased cell proliferation and downregulated stromal differentiation genes, including BMP2 in CO than in CN. Induction of IRS2 (a BMP2 regulator) mRNA expression by MPA led to activation of glucose metabolism in CO, with evidence of greater mRNA levels of GLUT6, GAPDH, PKM2, LDHA, and increased in GAPDH enzymatic activity. Concomitantly, MPA increased the mRNA expression of a fatty acid transporter, CD36 and lipid droplet formation in CO. MPA-mediated increase in glucose metabolism genes in CO was reversed with a progesterone receptor inhibitor, mifepristone (RU486), leading to a decreased proliferation. Our data suggests that PR signaling is aberrantly activated by MPA in CAFs isolated from endometrial tissues of obese women, leading to activation of IRS2 and glucose metabolism, which may lead to lower response and sensitivity to progesterone in obese women.

## 1. Introduction

Obesity [body mass index (BMI) > 30 kg/m^2^] is an established risk factor for developing endometrial cancer (EC) [1, 2]. Each 10 kg/m^2^ increase in BMI is associated with a 3-fold increase in risk [3, 4] with higher cancer incidence and mortality [2]. Obesity does not only affect EC progression, but also the response of patients to progestin treatment [5]. Medroxyprogesterone acetate (MPA), also known as progestin has been used conservatively to treat patients with EC due to its inhibitory effects on tumor growth [6–8]. However, complete response to oral MPA was reported to be less frequent among obese than lean EC patients, with increased risk for recurrence in obese patients [9, 10].

MPA exerts its inhibitory effect on endometrial tumor cells by acting through the progesterone receptor (PR) in the fibroblast cells in an *in vivo* mice model [11, 12]. However, the presence of cancer-associated fibroblasts (CAFs) has been associated with chemoresistance in many cancers including breast, lung and colorectal cancers [13, 14]. Similar observation was seen in hormonal therapy in which endometrial CAFs showed minimal response to MPA [15]. MPA inhibitory effect is accompanied by activation of stromal differentiation genes including bone morphogenetic protein 2 (BMP2), heart and neural crest derivatives expressed 2 (HAND2) and many others [16]. In particular, BMP2 is regulated by a PR direct target gene, insulin receptor substrate 2 (IRS2), which is required for stromal differentiation [17–19] to prevent growth and proliferation [20].

IRS2 plays a pivotal role in the activation of multiple downstream intracellular signaling cascades [18] to significantly alter glucose metabolism. IRS proteins are the primary mediators of glucose metabolism in most cell types [21, 22]. In particular, IRS2 has been shown to affect tumor progression and metastasis, largely through its ability to respond to the metabolic microenvironment [23, 24]. MPA has also been shown to affect glucose-insulin metabolism. Women who are prescribed with MPA as contraceptive were reported to have a higher plasma insulin, alkaline phosphatase and cortisol levels [25]. Contraceptive use of MPA in obese and overweight users especially, resulted in an increased in glucose levels than normal weight users [26], secondary to decreased glucose tolerance and enhanced insulin resistance observed in obese patients [27]. In an animal model, MPA has been shown to enhance lipid and protein synthesis during liver regeneration after liver injury in female rats [28].

However, it remains unknown if MPA modulate metabolism through PR and IRS2 signaling in CAFs isolated from obese women. The pathophysiology underlying the associations between obesity and the effectiveness of MPA remain incompletely understood. This study aims to investigate the effects of MPA in activating PR signaling pathway in endometrial CAFs isolated from obese and non-obese women.

In this study, we established several primary cultures of freshly isolated human endometrial fibroblast cells from obese and non-obese women EC tissues. MPA increased cell proliferation in CAFs from obese women compared to those from non-obese women. In CAFs isolated from obese women, MPA enhanced the expression of IRS2, which resulted in increased glucose metabolism and an increased in fatty acid uptake and storage. Treatment with PR inhibitor and IRS2 inhibitor, reversed the effects on mRNA expression and also resulted in an increased sensitivity towards MPA anti-proliferative effects in CAFs from obese women.

## 2. Materials and methods

### 2.1. Ethics statement

The study was approved by the Ethical Committee of the University of Malaya Medical Centre (MEC Ref. 865.19). Written informed consent was obtained from all participants.

### 2.2. Chemicals and reagents

Medroxyprogesterone acetate (MPA, Cat#M1629), and mifepristone (RU486, Cat#M8046) were obtained from Sigma-Aldrich, St. Louis, MO. NT157 (Cat#S8228) was obtained from SelleckChem (Houston, USA).

### 2.3. Endometrial cancer tissues and cells isolation

Tissues were obtained from eight women undergoing surgery to remove parts of their endometrium. These tissues were identified as Type I endometrial cancers. None of the subjects received any hormonal therapy for at least 6 months prior to surgery. Patients were categorized into two groups which are non-obese (BMI: 18.5–24.9 kg/m^2^) and obese (BMI > 30 kg/m^2^). About 1 g of tissues was subjected to stromal isolation using anti-fibroblast magnetic microbeads (Miltenyi Biotec, Cologne, Germany) as described previously [29]. The fibroblasts were categorized as CAFs isolated from non-obese (CN) and obese (CO) women and were labeled according to each individual patient. Apart from different category of BMI, both CN and CO have similar molecular expression of basal PR (S1 Table). Moreover, the expression of fibroblast (vimentin and alpha-smooth muscle actin) and epithelial (EpCAM and E-cadherin) markers in both group was also similar. All treatment with vehicle and MPA were performed in high glucose condition using phenol red-free DMEM/F12 media, which contained 18 mM glucose, supplemented with 10% charcoal-stripped FBS (Biowest, USA) and 1% penicillin/streptomycin (Life Technologies, USA). Experiment was performed with cultures of passage 12 and below to maintain the closest phenotype to its original excised tissue.

### 2.4. Quantitative real-time polymerase chain reaction (qRT-PCR)

Total RNA was extracted from cultured cells using TRIzol (Invitrogen, California, USA) and first-strand complementary DNA (cDNA) was synthesized by reverse transcription using RevertAid RT Reverse Transcription Kit (Cat#K1691, Thermo Scientific, USA) with 300 ng of total RNA. The characterization and RT-PCR of fibroblast cells were done according to previous methods [29]. For basal total PR mRNA expression, the gene expression was normalized to GAPDH gene and then compared to ECC-1, in which PR is expressed [30, 31]. For stromal differentiation genes, the gene expression of CN1-4 and CO1-4 treated with 10 nM MPA for 4 hours was normalized to GAPDH gene and then compared to vehicle. For metabolism genes, the gene expression of CN1-4 and CO1-4 treated for 6 hours with 10 nM MPA, 10 μM RU486 or 1 μM NT157 was normalized to 18s sRNA gene and then compared to vehicle. The 4- to 6- hour timepoint were chosen to reflect early response of MPA on stromal cells gene expression [32, 33]. Specific primers were listed in S2 Table. The heat map was constructed using GraphPad Prism. Data shown are representative of three independent experiments.

### 2.5. Total protein extraction and western blotting

Cells were seeded at 2x10^5^ cells/well in 6-well plates in complete media. Whole cell lysates were collected by scraping cells in cold lysis buffer containing 0.1% Triton-X, 0.1% sodium dodecyl sulfate (SDS), 50 mM Tris, 150 mM NaCl, 1x phosphatase and 1x protease inhibitors [29]. Protein concentrations were determined using Bradford assay (Biorad, CA, USA). The protein lysates were resolved and transferred as described previously [29]. Membranes were incubated overnight at 4°C with primary antibody against insulin receptor substrate 2 (1:500, Cat#ab134101, Abcam, USA) and then incubated with secondary peroxidase-conjugated goat anti-rabbit antibody (1:10,000, Cat#sc-2313, Santa Cruz Biotechnology, USA) for 1 h at room temperature. The blots were visualized using SuperSignal West Femto Maximum Sensitivity Substrate (Cat#34094, Thermo Fisher Scientific, USA) using gel documentation system (FluorChem M System, Protein Simple, USA). The blots were reprobed with mouse β-actin antibody (1:1000, Cat#sc-47778, Santa Cruz Biotechnology, USA) for a loading control.

### 2.6. 5-bromo- 2-deoxyuridine (BrdU) cell proliferation assay

The BrdU cell proliferation assay (Cat#6813, Cell Signaling Technology, MA, USA) was used to measure cell proliferation rate in CN and CO for 24 hours post MPA treatment, in the presence and absence of 10 μM RU486 or 1 μM NT157. BrdU assay detects BrdU incorporation into cellular DNA during cell proliferation. Fibroblast cells were seeded at a concentration of 2x10^3^ cells per well in the 96-well plate. The cells were incubated with 10 or 1000 nM MPA concurrent with 10 μl 1X BrdU solution for 24 hours, prior to fixation for 30 minutes with the Fixing Solution at room temperature. This was followed by incubation of 100 μl of 1X BrdU detection antibody for one hour at room temperature. The stained cells were washed and incubated with 100 μl of 1X HRP-conjugated secondary antibody solution for 30 minutes at room temperature. Post-washing, 100 μl of TMB substrate were added to each well. Once color changes occurred and stabilized, 100 μl of STOP solution were added to terminate the assay and plates were read within 15 minutes. Results were read using Spectramax M3 multimode plate reader (Molecular Devices) at an absorbance of 450 nm. The data were normalized to vehicle.

### 2.7. Glyceraldehyde-3-phosphate dehydrogenase (GAPDH) enzymatic assay

GAPDH enzyme activity was measured using calorimetric assay kits from Biomedical Research Service Center, SUNY (BRSC, Buffalo, NY). All samples of CN1-4 and CO1-4 were harvested using 1X Cell Lysis Solution. Protein concentration of the samples were assessed with Bradford assay and normalized to 1 mg/ml. GAPDH activity was measured using GAPDH enzyme assay kit (Cat#E-101). Briefly, 10 μl of sample or water (as blank) was incubated in 50 μl of GAPDH assay solution. After gentle agitation, the plate was kept in a non-CO_2_ incubator at 37°C for 60 minutes. All experiments were terminated by adding 50 μl of 3% acetic acid and the plate was read at O.D._492 nm_ with a spectrometer. Blank reading was subtracted from the sample reading. GAPDH activity in IU/L unit was determined by multiplying O.D by 16.98.

### 2.8. Fatty acid oxidation (FAO) enzymatic assay

FAO enzyme activity was measured using calorimetric assay kits from Biomedical Research Service Center, SUNY (BRSC, Buffalo, NY). All samples of CN1-4 and CO1-4 were harvested using 1X Cell Lysis Solution. Protein concentration of the samples were assessed with Bradford assay and normalized to 1 mg/ml. FAO activity was measured using FAO enzyme assay kit (Cat#E-141). 50 μl of FAO assay solution or control solution was added to 10 μl of protein sample. After gentle agitation, the plate was kept in a non-CO_2_ incubator at 37°C for 60 minutes. All experiments were terminated by adding 50 μl of 3% acetic acid and the plate was read at O.D._492 nm_ with a spectrometer. Blank reading was subtracted from the sample reading. For FAO assay, the subtracted O.D. represents the fatty acid oxidation of the sample.

### 2.9. Oil red O staining

Oil red O powder (Cat#O0625, Sigma-Aldrich, USA) was dissolved in 2-propanol (0.5%). The stock was then diluted to 0.3% oil red O solution with distilled H_2_O and filtered through 0.22-μm filter. Fibroblast cells were seeded in 24-well plate (1E4/well) overnight. After treatment with 10 nM MPA for 24 h, the treated cells (CN 2,4 and CO 1,4) were washed with ice cold PBS and incubated with 60% isopropanol for 5 min. The coverslips were left air-dried before staining with Oil Red O dye for 15 min. Finally, the stained cells were washed three times with Millipore water, and counterstained with hematoxylin for 30 s and washed with PBS for three times. The cells were observed with a Nikon Eclipse E200 microscope (Nikon Instruments, Japan). Experiments were repeated twice and the representative data were shown. The stained lipid droplets were quantified using ImageJ software.

### 2.10. Statistical analysis

Statistical analysis that assessed the differences between the means of two groups was performed using Student’s *t*-test with GraphPad Prism software (GraphPad Prism version 7, USA). A P-value <0.05 was considered statistically significant.

## 3. Results

### 3.1. MPA induced cell proliferation in CAFs of obese women by decreasing the expression of stromal differentiation genes

**Fig 1:**
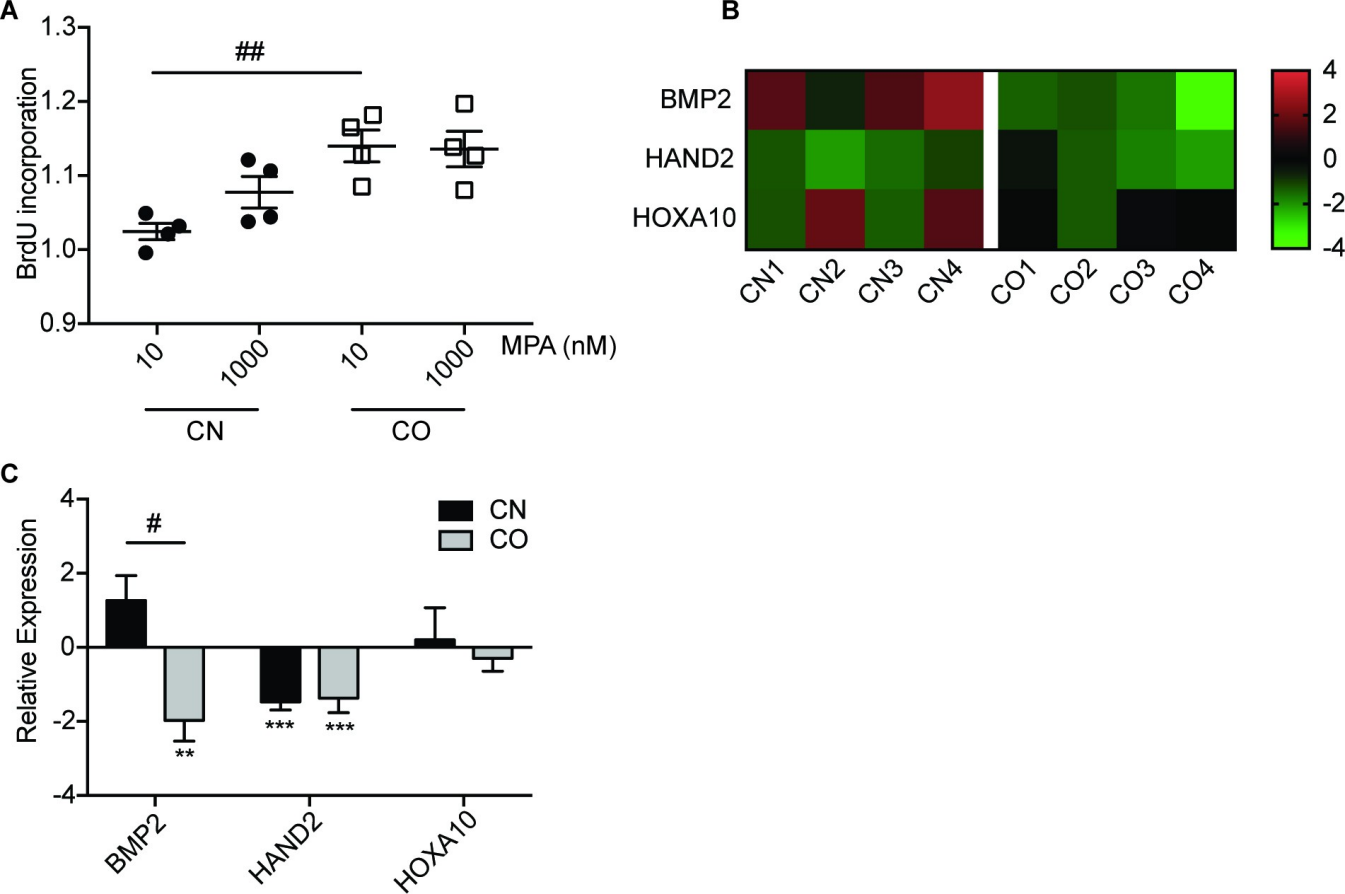
MPA induced cell proliferation in CAFs of obese women by decreasing the expression of stromal differentiation genes. (A) CAFs from non-obese (CN) and obese (CO) women were treated with 10 and 1000 nM MPA for 24 h, before measuring their proliferation using BrdU cell proliferation assay. The data was normalized to vehicle and the means of the data were shown. (B) The heatmap shows the expression of stromal differentiation genes. The bottom of the heatmap is labeled with cells CN1-4 and CO1-4, while the row is labeled with genes. CN and CO were treated with 10 nM MPA for 4 hours, before RNA isolation for quantitative real-time PCR analysis of stromal differentiation genes. The data was normalized to GAPDH followed by the value from cells treated with the vehicle. (C) The mean expression values of CN and CO from (B) were shown. Bar graphs represent mean ± SEM. **P<0.01, ***P<0.001, comparing MPA to vehicle. #P<0.05, ##P<0.01, comparing CO to CN. Data shown are representative of three independent experiments.

One of MPA functions in fibroblast cells is to inhibit cell growth [34]. Interestingly, both CAFs from obese (CO) and non-obese (CN) endometrial cancer tissues demonstrated some resistance to MPA’s anti-proliferative effects, as shown in BrdU proliferation assay (Fig 1A). There was a slight increase of proliferation (8%) in CN cells after being treated with 1000 nM MPA when compared to vehicle-treated cells. CAFs from obese women (CO) showed a greater increase in cell proliferation at 14%, when treated with 10 nM and 1000 nM MPA. Analysis with annexin V/7-AAD apoptosis assay showed no significant apoptotic effect in CN and CO following MPA treatment (S1 Fig). These cells were subjected to 10 nM MPA treatment for 4 h, before examining the expression of stromal differentiation genes. As shown in the heatmap of mRNA level in individual cell (Fig 1B), an overall downregulation of BMP2, HAND2, and homeobox A10 (HOXA10) gene expression was observed in MPA-treated CO cells, whereas some CN cells responded to MPA-induced BMP2 and HOXA10 mRNA expression. BMP2 and HOXA10 were differentially downregulated in CO cells (-1.97-fold and -0.3-fold, respectively) when compared to those in CN cells (Fig 1C). These data suggest that CAFs from obese women (CO) responded differently to MPA with increased cell proliferation and downregulation of stromal differentiation gene expression, when compared to those from non-obese women (CN).

### 3.2. MPA induced IRS2 and increased glucose metabolism in CAFs of obese women

**Fig 2:**
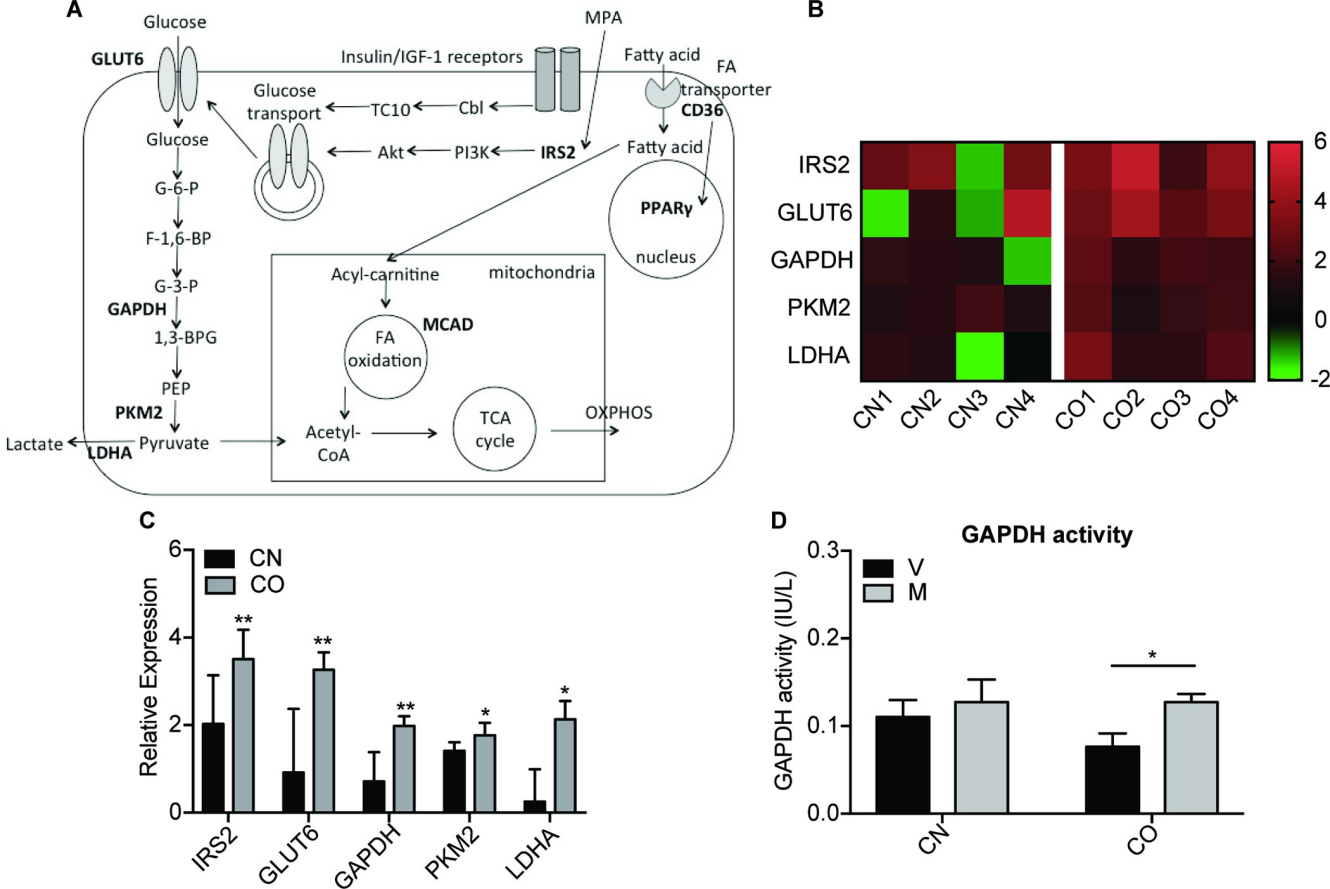
MPA induced IRS2 and increased glucose metabolism in CAFs of obese women. (A) Diagram highlighting genes involved in glycolytic and fatty acid metabolism. (B) The heatmap shows the expression of metabolic genes in CN and CO after 6 h treatment of MPA. The bottom of the heatmap is labeled with cells CN1-4 and CO1-4, while the row is labeled with genes. (C) The mean expression values of IRS2 and glycolytic genes for CN1-4 and CO1-4. The data was normalized to housekeeping gene 18S rRNA. (D) GAPDH enzymatic activity in CN1-4 and CO1-4 were measured with GAPDH assay kit 24 h post MPA treatment, and normalized to the protein concentration of the samples. V: vehicle, M: MPA; *P<0.05, **P<0.01, comparing MPA treatment to vehicle for CN1-4 and CO1-4. Data represent the mean ± SEM., and are representative of three independent experiments.

The differential proliferative profiles of CO and CN cells may be due to the metabolic states of their tissue of origin. First, we examined the key genes involved in glucose metabolism including IRS2, which is a direct PR target gene (Fig 2A). Heatmap analysis showed that all CO cells responded to MPA treatment with upregulation of IRS2, as well as glucose transporter 6 (GLUT6), glyceraldehyde-3-phosphate dehydrogenase (GAPDH), pyruvate kinase M2 (PKM2), and lactate dehydrogenase A (LDHA) genes expression (Fig 2B). However, these genes were not upregulated to the same extent in CN cells, except for IRS2. When analyzed collectively (Fig 2C), MPA significantly induced the mRNA expression of IRS2 (3.51-fold), GLUT6 (3.26-fold), GAPDH (1.98-fold), PKM2 (1.77-fold), and LDHA (2.13-fold) in CO cells. While not significant, modest increase of IRS2 and PKM2 gene levels was observed in CN cells. MPA also induced glycolytic enzyme activity in CO (67%, P<0.05) but not in CN, when compared to vehicle, although there was no significant difference between CO with CN (Fig 2D). These data indicate that MPA significantly enhanced glycolytic metabolism in CO, which may be used as bioenergetics to fuel for its growth.

### 3.3. MPA increased expression of CD36 and lipid droplet formation in CAFs of obese women

**Fig 3:**
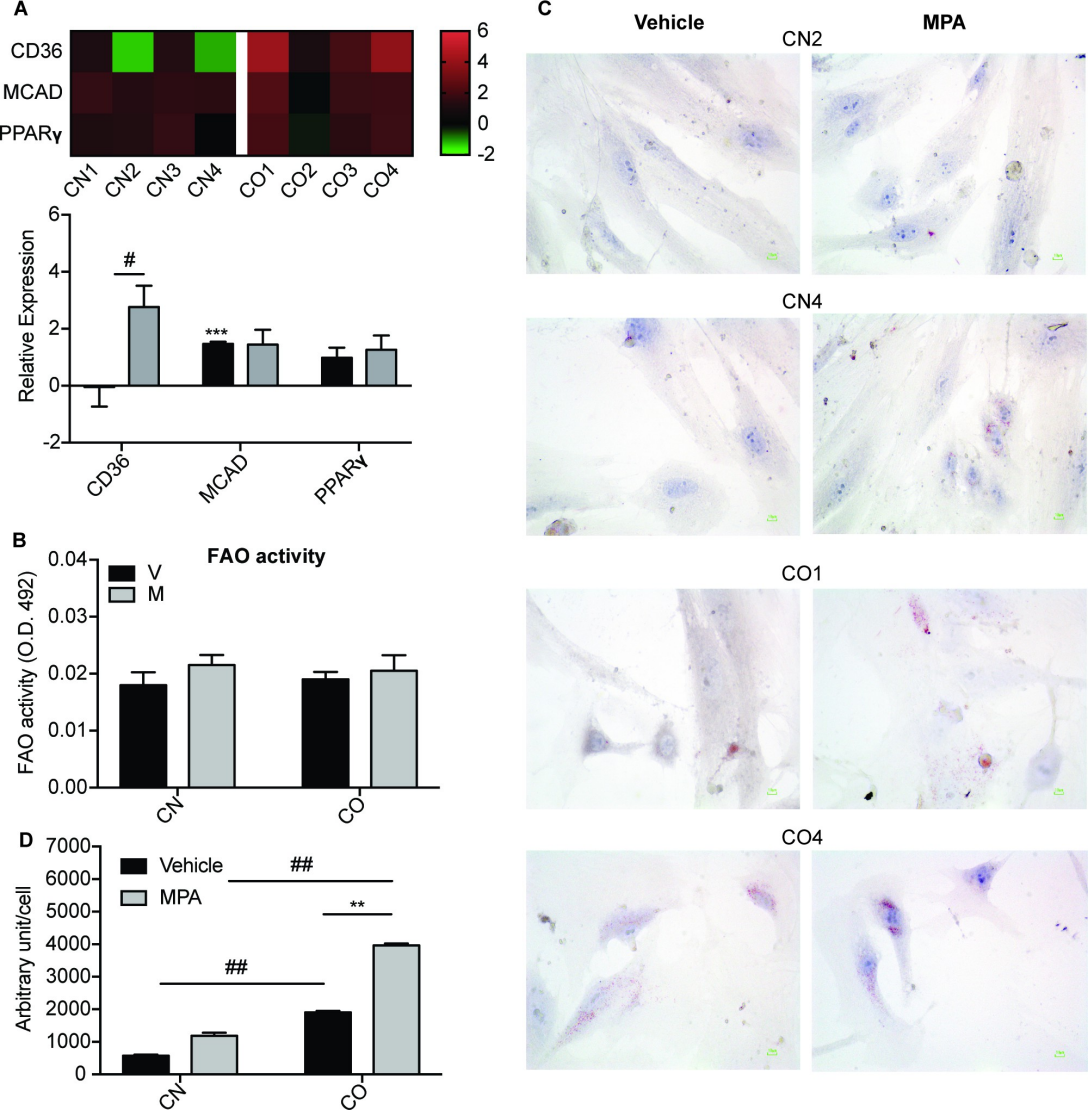
MPA increased expression of CD36 and lipid droplet formation in CAFs of obese women. (A) The heatmap shows the expression of metabolic genes in CN and CO after 6 h treatment of MPA, and the mean expression values of genes involved in fatty acid metabolism in CN1-4 and CO1-4. The data was normalized to housekeeping gene 18S rRNA. (B) Fatty acid oxidation (FAO) activity in CN1-4 and CO1-4 was measured with FAO assay kit 24 h post MPA treatment, and normalized to the protein concentration of the samples. Data shown are mean ± SEM of duplicates. (C) Lipid droplet formation was measured in CN (2, 4) and CO (1, 4) after treatment with 10 nM MPA for 24 h. The coverslips were stained with Oil Red O dye and counterstained with haematoxylin, before analyzing with light microscopy at 40X magnification. Scale, 200 μM. Images were analyzed using ImageJ software. Representative images were shown for each CN and CO. (D) Bar graphs showing quantification of lipid droplet formation. Data shown are the mean ± SEM of three different spots in a coverslip. V: vehicle, M: MPA; **P<0.01, ***P<0.001, comparing MPA treatment to vehicle. #P<0.05, ##P<0.01 comparing CO to CN.

Interestingly, the change in glucose metabolism induced by MPA was accompanied with changes in fatty acid metabolism. Expression of genes involved in fatty acid transport (CD36), fatty acid oxidation (medium-chain acyl-CoA dehydrogenase, MCAD), and fatty acid storage (peroxisome proliferator-activated receptor, PPARγ) were measured after 6 h of MPA treatment. MPA significantly upregulated the expression of CD36 mRNA in CO (2.76-fold) but not in CN (-0.04-fold, P<0.05), with no significant difference for MCAD and PPARγ mRNA levels (Fig 3A). Yet, FAO activity remained similar in both cells after treated with MPA in comparison to control cells (Fig 3B). Interestingly, oil red O staining revealed a significant accumulation of basal lipid droplets in CO than in CN cells (vehicle treated) (Fig 3C and 3D). Treatment with MPA for 24 h resulted in a further increase in lipid droplet formation in CO (2.2-fold, P<0.01 vs vehicle) (Fig 3C and 3D). Such accumulation of lipid droplets was not observed in CN, with more than 3.3-fold increased in lipid droplet formation observed in MPA-treated CO vs CN cells (P<0.01). Taken together, these data suggest that upregulation of CD36 gene expression by MPA in CO cells may results in increased of fatty acid uptake, leading to enhanced fatty acid storage in lipid droplets which can be used for structural membrane formation during proliferation.

### 3.4. Activation of genes related to glucose and fatty acid metabolism is progesterone receptor-dependent

**Fig 4:**
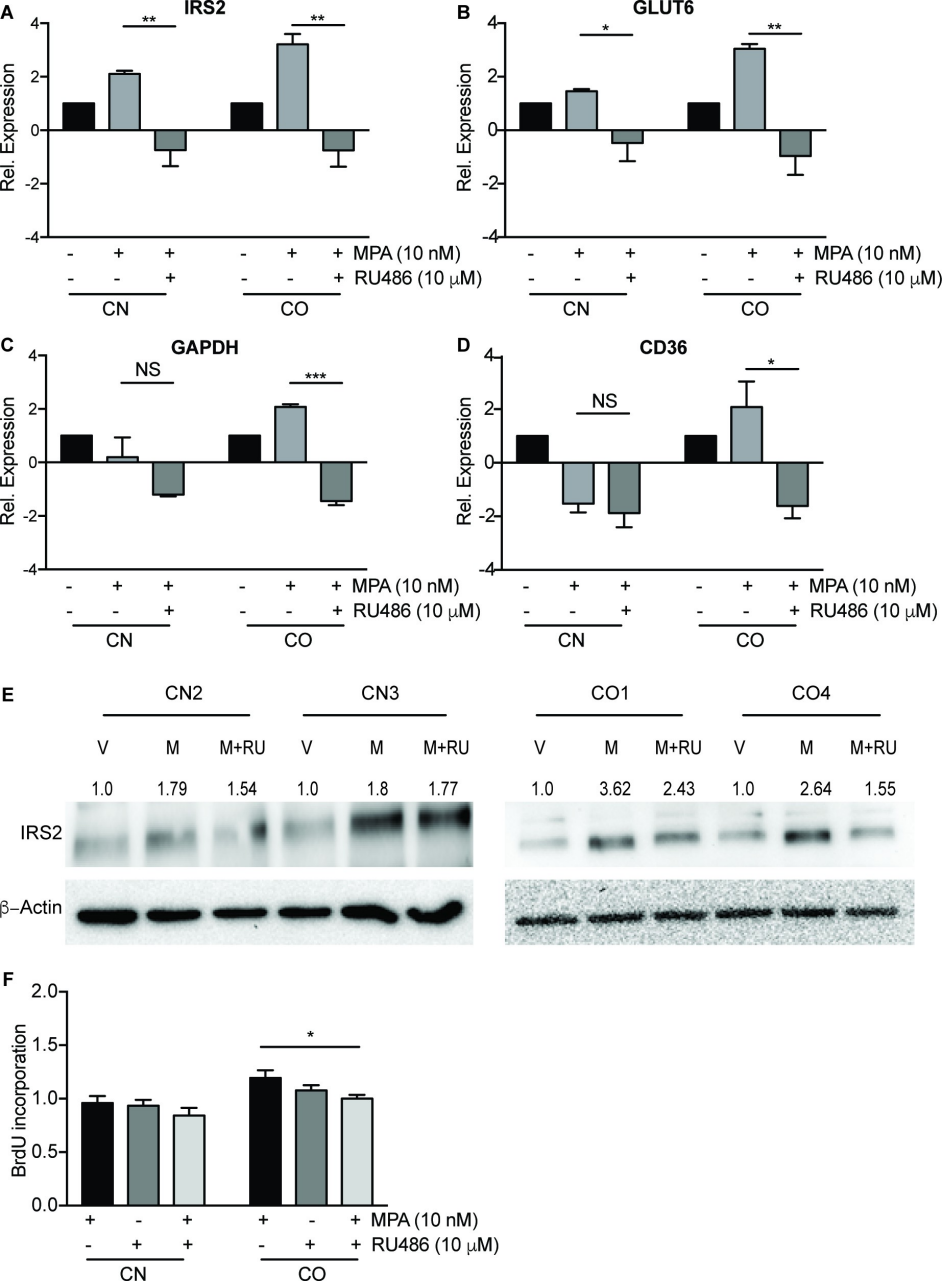
Activation of genes related to glucose and fatty acid metabolism is progesterone receptor-dependent. Gene expression of metabolic genes IRS2, GLUT6, GAPDH and CD36 in CN1-4 and CO1-4 after treatment with 10 nM MPA and 10 μM RU486 (A-D) for 6 h. (E) IRS2 protein level in CN and CO 24 h after treatment in vehicle (V), 10 nM MPA (M), and MPA+10 μM RU486 (M+RU) treated cells, evaluated using Western blotting. (F) CN and CO were treated with 10 nM MPA, in combination with PR inhibitor, RU486 for 24 h, before measuring their proliferation using BrdU cell proliferation assay. The data was normalized to vehicle and the means of the data were shown. Bar graphs represent mean ± SEM. NS: non-significant. *P<0.05, **P<0.01, ***P<0.001. Data shown are representative of three independent experiments.

It remains unknown if the increased glucose metabolism and fatty acid storage observed in CO cells were attributed by the action of MPA. Only IRS2 has been reported as PR target gene [17, 19]. Hence, we determined the expression of IRS2, GLUT6, GAPDH and CD36 mRNA in MPA-treated cells in the presence of progesterone receptor inhibitor RU486. Expectedly, IRS2 mRNA expression was downregulated by -0.8-fold (P<0.01) in CO cells treated with the combination treatment, indicating that IRS2 induction was due to PR activation by MPA (Fig 4A). Interestingly, RU486 also decreased the expression of GLUT6 (-1.0-fold, P<0.01), GAPDH (-1.4-fold, p<0.001) and CD36 (-1.6-fold, P<0.05), when compared to MPA treatment alone (Fig 4B–4D). Similarly, MPA treatment for 24 h increased IRS2 protein level by 2.6- to 3.6-fold in CO, while the addition of RU486 attenuated IRS2 protein level by 30–40%. Meanwhile, MPA treatment increased IRS2 protein level in CN, but to a lesser degree compared to those of CO (1.8-fold) and co-treatment with RU486 only decreased IRS2 protein level by 2–14% in CN (Fig 4E). Subsequently, the combination treatment of MPA and RU486 resulted in a significant 16% (P<0.05) decrease in cell proliferation in CO cells when compared to MPA alone, with no change in CN cells (Fig 4F). This indicates that PR activation by MPA is required for the induction of glucose metabolism in CO cells. When PR is inhibited, glucose metabolism is suppressed leading to reversal of MPA-induced cell proliferation.

### 3.5. MPA-induced IRS2 mediates activation of genes related to glucose and fatty acid metabolism

**Fig 5:**
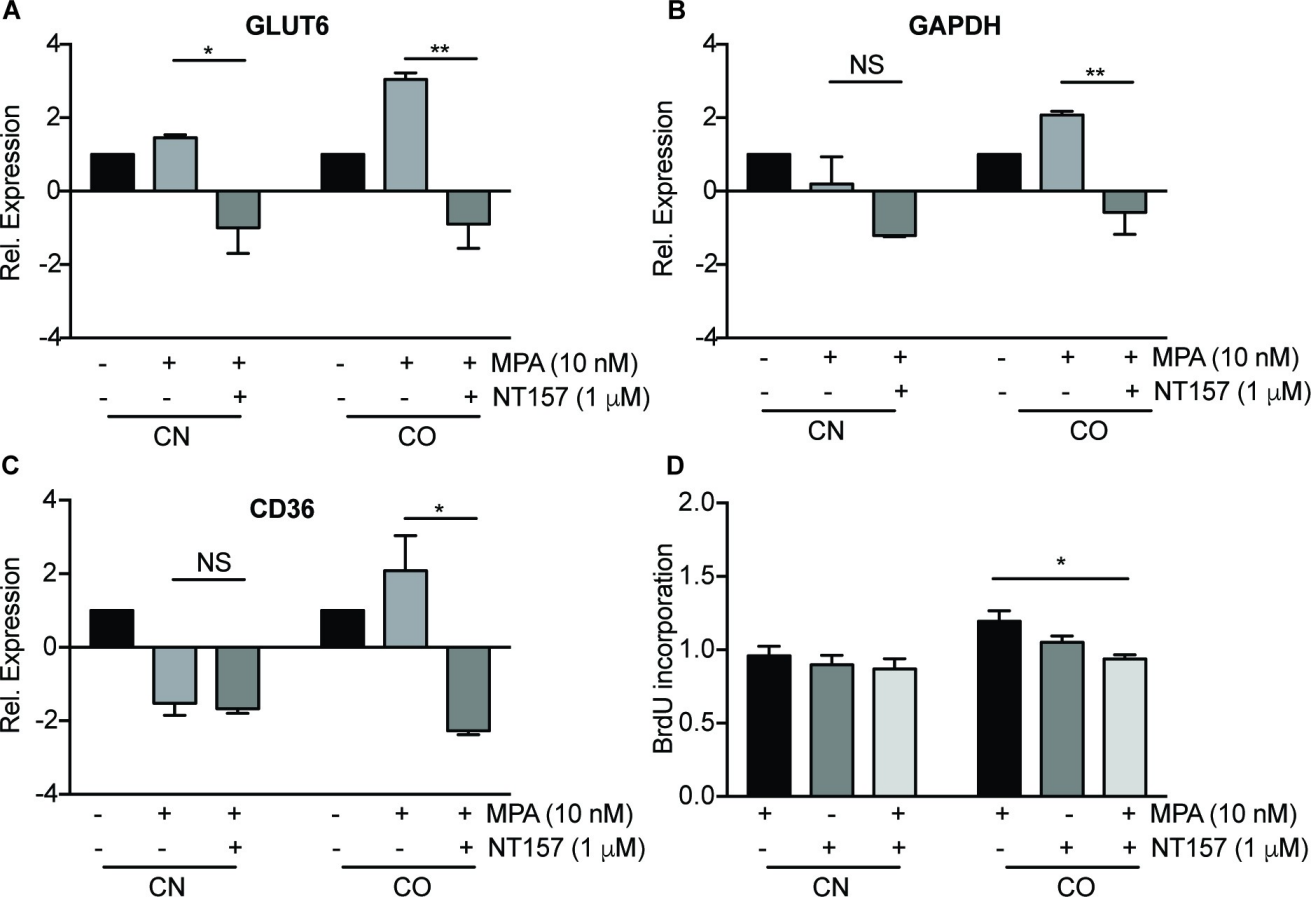
MPA-induced IRS2 mediates activation of genes related to glucose and fatty acid metabolism. Gene expression of GLUT6, GAPDH and CD36 in CN1-4 and CO1-4 after treatment with 10 nM MPA and 1 μM NT157 (A-C) for 6 h. (D) CN and CO were treated with 10 nM MPA, in combination with IRS2 inhibitor, NT157 for 24 h, before measuring their proliferation using BrdU cell proliferation assay. The data was normalized to vehicle and the means of the data were shown. Bar graphs represent mean ± SEM. NS: non-significant. *P<0.05, **P<0.01. Data shown are representative of three independent experiments.

It is also possible that the induction of GLUT6, GAPDH and CD36 expression occurs due to increased IRS2 via PR signaling [35]. Indeed, co-treatment of MPA and IRS2 inhibitor (NT157) significantly reduced the mRNA expression of GLUT6 (-0.9-fold, P<0.01), GAPDH (-0.6-fold, P<0.01) and CD36 (-2.3-fold, P<0.05) (Fig 5A–5C). These data suggest that activation of these genes was dependent on IRS2. Subsequently, combination treatment of MPA with IRS2 inhibitor, NT157 also lead to lesser cell proliferation in CO (21%, P<0.05) compared to MPA alone (Fig 5D). There was also no significant change observed in CN following treatment. Our data indicates that MPA modulates glucose metabolism through IRS2 signaling in CAFs isolated from obese women. IRS2 inhibition led to reversal of MPA-induced cell proliferation in these cells.

## 4. Discussion

Obesity and its associated metabolic disturbances are well-known driver for EC prevalence and the lack of the disease response to MPA [9, 10]. Recent studies indicated that normal fibroblasts may be a target for MPA, in its action to inhibit tumor growth [11, 12]. However, it remains unknown if metabolic states of fibroblasts from obese EC patients affect MPA actions. Hence, in this study, we compared the effects of MPA on CAFs isolated from EC tissues of obese (CO) and non-obese (CN) patients. Our results showed that these cells exhibited varied induction of stromal differentiation genes by MPA. Lower expression of the differentiation genes in CO correlated with a higher proliferative rate after treatment with MPA. Compared to CN, there was a higher induction of IRS2 mRNA expression in CO, resulting in activation of glucose metabolism via enhanced GLUT6 expression and GAPDH enzymatic activity. Further, MPA also increased the expression of the fatty acid transporter, CD36 leading to greater fatty acid storage in lipid droplets in CO. Treatment with PR and IRS2 inhibitors individually led to decreased IRS2 and its downstream signaling mRNA expression in CO, which subsequently inhibited CO cells proliferation. These findings support the notion that MPA modulates metabolic phenotype of fibroblasts through IRS2 signaling pathway for growth and survival.

Obesity-associated systemic signals serve dual roles for cancer metabolism. In one aspect, these factors have been shown to augment oncogene-induced metabolic reprogramming by supplying ample substrates and supportive growth factor signaling in many cancers [36, 37]. Evidently, obesity has been shown to enhance the rate of metabolic reprogramming in tumors isolated from obese mice with increased in glycolytic intermediates and lipid biosynthesis hence promoting endometrial [38] and breast tumorigenesis [39]. Mice fed with high-energy diet displayed metabolic stress that resulted in higher concentrations of insulin and IGF-1, which mediate ovarian cancer tumor growth [40]. Metabolic alteration has also been found in obese cancer patients including hyperinsulinemia and elevated serum level of IGF-1 [40–42]. These findings suggest that obesity-induced metabolic disturbances are central to pathogenesis of hormonal responsive cancer. In another aspect, obesity-associated signals also significantly contribute to the restructuring of metabolic landscape of tumor microenvironment to drive tumorigenesis. Obese mice fed with high-fat diet showed altered fatty acid partitioning in the tumors, impairing CD8+ T cell infiltration function [43]. Metabolically, abundance of lipids from adipocytes in the tumor microenvironment has also been shown to support tumor progression and growth, with cancer-associated adipocytes ability to release fatty acids through lipolysis which are used by tumor cells for energy production through β-oxidation [44]. Our study further demonstrated that patient’s BMI can affect the metabolic landscape and proliferative states of CAFs, through modulation by MPA therapy.

While it is now established that obesity directly alter the metabolism of tumor and other neighboring cells to promote malignancy, the effect of obesity on the metabolism of fibroblast cells is less understood. We have demonstrated that CAFs derived from obese patients respond differently to MPA by activating PR and IRS2 signaling, leading to increased glycolytic metabolism, lipid uptake and storage. IRS2, a direct PR target gene, controls various downstream signaling cascades primarily affecting glucose metabolism for growth and survival of tumor cells [21, 45]. Parallel to this, dose-dependent proliferative effect was not apparent in CO cells following MPA treatment, potentially due to the saturation of pyruvate dehydrogenase activity resulting from high glycolytic flux [46], secondary to IRS2 activation. Our findings are in line with the literature, in which upregulation of IRS2 promoted glucose uptake and glycolysis in mammary tumor cells [24] and chronic myelogenous leukemia cells [47]. In obese patients, increased IRS2 expression is shown to be associated with steatohepatitis and altered lipid metabolism [48]. These findings suggest the role of IRS2 in promoting both glucose and lipid metabolism which may lead to cancer progression.

Previously, the relationship between PR and IRS2 has been established. The crosstalk of progesterone and PR with IGF-1 signaling via IRS2 promote growth of breast [18] and cervical [19] cancer cells. Meanwhile, in normal human endometrial stromal cells, PR-regulated expression of IRS2 induced glucose uptake and decidual markers expression including HAND2 to promote decidualization [49]. However, in our CAFs model, despite an increased in IRS2 expression, there is a decreased in HAND2 expression, parallel to previously reported study which demonstrated loss of HAND2 expression in the stromal of atypical hyperplasia and endometrioid adenocarcinomas compared to normal fibroblasts [50]. In this study, we have demonstrated that PR-IRS2 axis can also be preferentially activated by MPA in CAFs derived from obese patients, and not in cells from non-obese patients, suggesting the role of PR-IRS2 signaling in the tumor microenvironment of obese patients. Meanwhile, independent of obesity, the presence of IRS2 does not only affect tumorigenesis but its detrimental effects have also been highlighted in other therapies. The presence of IRS2 inhibited the ability of IRS1 to sensitize myeloblast-like and breast cancer cells to taxol, etoposide, and vincristine therapy [51, 52] and silencing of IRS2 potentiates the effects of ruxolitinib in myeloproliferative neoplasms cells [53]. Taken together, the presence of IRS2, a candidate driver oncogene, may modulate metabolic activities and response to therapy in cancer patients, particularly in those with high BMI.

Significant effort has been invested to translate the understanding of obesity-associated changes into therapeutic strategies for cancer. Clinically, treatment outcome has been shown to be affected by the obesity state of patients, including MPA. Obese patients have demonstrated lower complete response to oral MPA (33%) than lean (85%) EC patients, and have an increased risk of recurrence [9, 10]. The underlying mechanism is unknown, but we postulate that it may be related to how fibroblasts from obese women respond to MPA through IRS2. The decreased response to MPA in CAFs secondary to its ability to enhance the glycolytic-lipogenic pathway in obesity strongly suggest that BMI of patients need to be carefully considered when treating patients with MPA therapy. It was reported that EC patients that undergo MPA therapy has an increased in blood glucose and serum insulin levels [54], and decreased total cholesterol and serum triglycerides [55]. However, the effects of MPA specifically on glucose and fatty acid metabolism genes including GLUT6 and CD36 in these patients are still unknown. Moreover, it has been shown in a Phase II study that metformin inhibits EC recurrence after MPA therapy by altering insulin resistance which subsequently improves the hormonal therapy outcomes of obese EC patients [56, 57]. Parallel to this, the use of PR inhibitor, RU486, in clinical setting has been proven beneficial especially in patients suffering from metabolic syndrome [58]. RU486 has been demonstrated to ameliorates obesity and metabolic disturbances [59] which suggest the potential therapeutic application of this agent for obese patients. With MPA ability to modulate genes controlling glycolytic-lipogenic processes, these metabolic pathways appear to impact the outcome of treatment and disease progression in obese women with endometrial cancer. Therefore, therapeutics targeting the glycolytic-lipogenic pathway may improve survival rates for these subsets of patients [37].

## 5. Conclusion

Our study established a critical role of MPA in modulating PR and IRS2 signaling pathway for growth and survival in CAFs isolated from obese women. MPA induced activation of IRS2 promotes glucose metabolism, and fatty acid uptake and storage to meet the energy demand during cell proliferation. These data showed that CAFs isolated from obese women are highly metabolically active in the presence of MPA, which may lead to lower response and sensitivity to progesterone in obese women. These findings provide more understanding on how MPA therapy can be affected by the obesity state of the patients. Future studies are required to delineate the effect of MPA therapy in obese EC patients with metabolic syndrome such as hyperinsulinemia and diabetes.

## Supporting information

S1 Table Characterization of cancer-associated fibroblasts isolated from non-obese (CN) and obese (CO) endometrial cancer patients using RT-PCR (relative expression log2 ratio). (DOCX)

S2 Table Primers for RT-PCR. (DOCX)

S1 Fig MPA does not cause significant apoptosis effect in CN and CO. Percentage of necrotic and apoptotic cells in CN2 and CO4 was measured using annexin V/7-AAD apoptosis assay following treatment with vehicle, 10 nM or 1000 nM MPA for 72 hours. Data shown are the mean ± SEM of triplicates.
(TIF)
