# Correction: MPA alters metabolic phenotype of endometrial cancer-associated fibroblasts from obese women via IRS2 signaling

**DOI:** 10.1371/journal.pone.0315116

**Published:** 2024-12-03

**Authors:** Intan Sofia Omar, Amira Hajirah Abd Jamil, Noor Azmi Mat Adenan, Ivy Chung

There are errors in the Funding statement. The correct Funding statement is as follows: This research was supported by Malaysian Ministry of Education (MOE) through Fundamental Research Grant Scheme (FRGS, FP029-2014B; grant code: FRGS/2/2014/SKK01/UM/02/1 to I.C.) and the University of Malaya High Impact Research Grant (UM.C/625/HIR/MED/12 to I.C.). The funders had no role in study design, data collection and analysis, decision to publish, or preparation of the manuscript.
