# Supplementary material for: MPA alters metabolic phenotype of endometrial cancer-associated fibroblasts from obese women via IRS2 signaling
Source: PLoS One. 2022 Jul 11;17(7):e0270830. doi: 10.1371/journal.pone.0270830 (PMC9273069; doi:10.1371/journal.pone.0270830)
Supplement: S1 Table — (DOCX) [file pone.0270830.s001.docx]

| **Cells** | **Category** | **BMI** | **PR**  **(log2 ratio)** | **α-SMA**  **(log2 ratio)** | **Vimentin**  **(log2 ratio)** | **EpCAM**  **(log2 ratio)** | **E-cadherin**  **(log2 ratio)** |
| --- | --- | --- | --- | --- | --- | --- | --- |
| CN1 | Non-obese | 19.3 | 2.49 | 6.77 | 498.39 | 0.59 | 0.69 |
| CN2 |  | 23.4 | 1.70 | 7.08 | 32609.40 | 3.46 | 2.92 |
| CN3 |  | 23.7 | 0.43 | 117.89 | 9.69 | 0.39 | 0.46 |
| CN4 |  | 24.4 | 11.05 | 20.40 | 144.70 | 0.04 | 0.02 |
| CO1 | Obese | 31.0 | 1.34 | 73.59 | 35511.00 | 1.13 | 0.63 |
| CO2 |  | 33.1 | 1.71 | 1251.50 | 3130.50 | 1.29 | 0.23 |
| CO3 |  | 33.1 | 0.63 | 35.12 | 84.44 | 1.18 | 0.54 |
| CO4 |  | 34.9 | 1.20 | 2.37 | 174.57 | 0.06 | 0.13 |

S1 Table. Characterization of cancer-associated fibroblasts isolated from non-obese (CN) and obese (CO) endometrial cancer patients using RT-PCR (relative expression log2 ratio).
