# Supplementary material for: MPA alters metabolic phenotype of endometrial cancer-associated fibroblasts from obese women via IRS2 signaling
Source: PLoS One. 2022 Jul 11;17(7):e0270830. doi: 10.1371/journal.pone.0270830 (PMC9273069; doi:10.1371/journal.pone.0270830)
Supplement: S2 Table — (DOCX) [file pone.0270830.s002.docx]

S2 Table. Primers for RT-PCR

| Gene | Direction | Sequence (5’ to 3’) |
| --- | --- | --- |
| 18S rRNA | Forward | GTAACCCGTTGAACCCCATT |
|  | Reverse | CCATCCAATCGGTAGTAGCG |
| BMP2 | Forward | TTCGGCCTGAAACAGAGACC |
|  | Reverse | CCAAAGATTCTTCATGGTGGAAGC |
| CD36 | Forward | ATGTAACCCAGGACGCTG |
|  | Reverse | GTCGCAGTGACTTTCCCA |
| GAPDH | Forward | CCCATCACCATCTTCCAGGAG |
|  | Reverse | GTTGTCATGGATGACCTTGGC |
| GLUT6 | Forward | GCCCGGACTACGACACCT |
|  | Reverse | AGCTGAAATTGCCGAGCAC |
| HAND2 | Forward | AGAGGAAGAAGGAGCTGAACGA |
|  | Reverse | CGGCCTTTGGTTTTCTTGTCG |
| HOXA10 | Forward | CTCACGGCAAAGAGTGGTC |
|  | Reverse | ATCCGGTTTTCTCGATTCAT |
| IRS2 | Forward | ACCTACGCCAGCATTGACTT |
|  | Reverse | CCTTGTTGGTGCCTCATCTAA |
| LDHA | Forward | TTGGTCCAGCGTAACGTGAAC |
|  | Reverse | CCAGGATGTGTAGCCTTTGAG |
| MCAD | Forward | GGAAGCAGATACCCCAGGAAT |
|  | Reverse | AGCTCCGTCACCAATTAAAACAT |
| PKM2 | Forward | CAGCCAAAGGGGACTATCCT |
|  | Reverse | TTCCTCAAATAATTGCAAGTGG |
| PPARγ | Forward | GAGGGCCAAGGCTTCATGA |
|  | Reverse | AGGCTTTCGCAGGCTCTTTAG |
