# Supplementary figures and images for: MPA alters metabolic phenotype of endometrial cancer-associated fibroblasts from obese women via IRS2 signaling
Source: PLoS One. 2022 Jul 11;17(7):e0270830. doi: 10.1371/journal.pone.0270830 (PMC9273069; doi:10.1371/journal.pone.0270830)

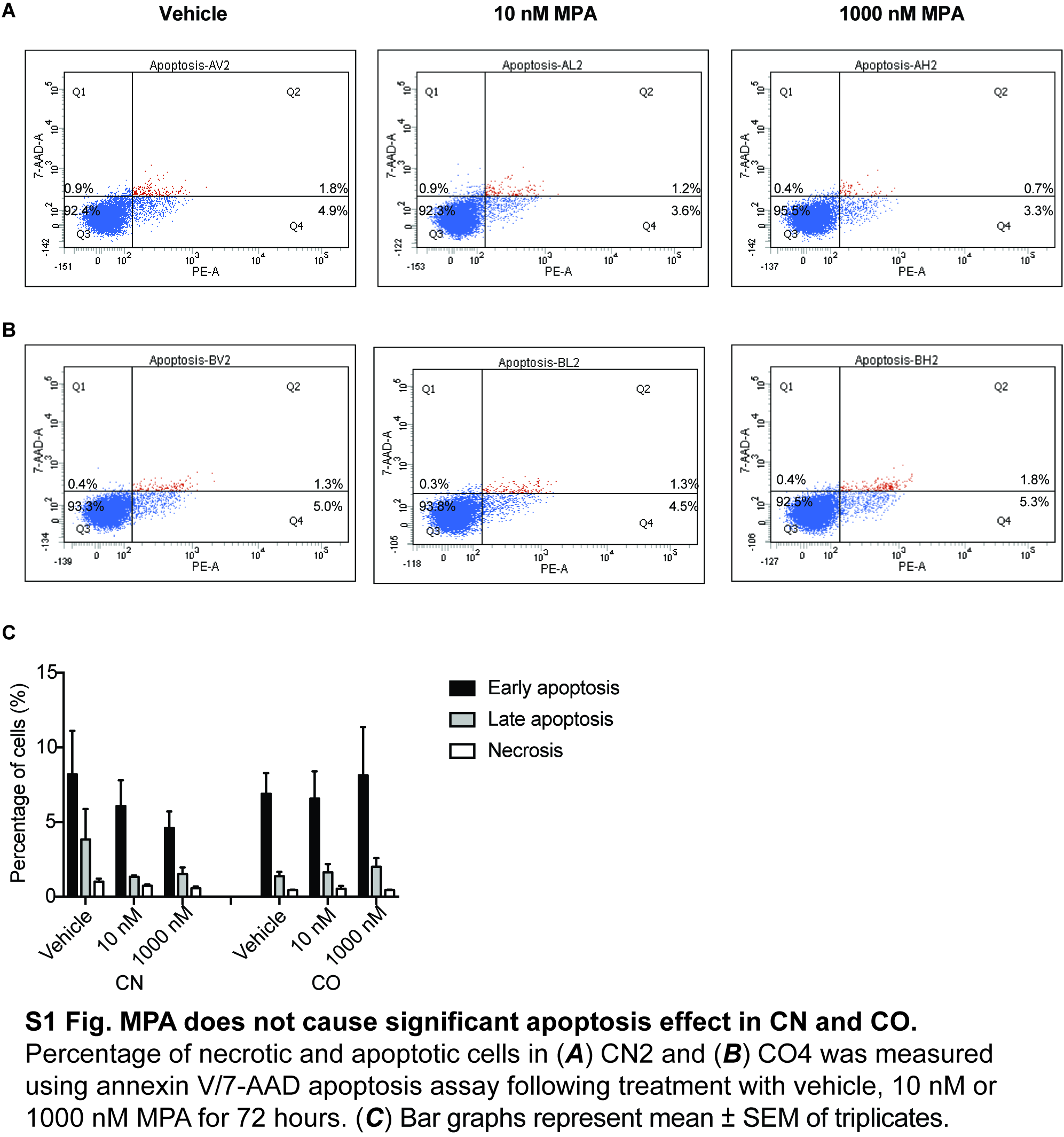

Supplement: S1 Fig — Percentage of necrotic and apoptotic cells in CN2 and CO4 was measured using annexin V/7-AAD apoptosis assay following treatment with vehicle, 10 nM or 1000 nM MPA for 72 hours. Data shown are the mean ± SEM of triplicates. (TIF) [file pone.0270830.s003.tif]
